# Supplementary material for: Comprehensive Analysis of the Hsp20 Gene Family in Canavalia rosea Indicates Its Roles in the Response to Multiple Abiotic Stresses and Adaptation to Tropical Coral Islands
Source: Int J Mol Sci. 2022 Jun 8;23(12):6405. doi: 10.3390/ijms23126405 (PMC9223760; doi:10.3390/ijms23126405)
Supplement: Supplementary file 1 [file ijms-23-06405-s001.zip › ijms-1745056-supplementary.pdf]

Supplementary Material Information

Comprehensive analysis of the *Hsp20* gene family in *Canavalia rosea* indicates its roles in the response to multiple abiotic stresses and adaptation to tropical coral islands

Mei Zhang [1,2,*], Shuguang Jian [2], Zhengfeng Wang [1,2,3,*]

[1] Guangdong Provincial Key Laboratory of Applied Botany&South China Agricultural Plant Molecular Analysis and Genetic Improvement, South China Botanical Garden, Chinese Academy of Sciences, Guangzhou 510650, China; zhangmei@scbg.ac.cn (M.Z.)
[2] CAS Engineering Laboratory for Vegetation Ecosystem Restoration on Islands and Coastal Zones, South China Botanical Garden, Chinese Academy of Sciences, Guangzhou 510650, China; jiansg@scbg.ac.cn (S.J.)
[3] Southern Marine Science and Engineering Guangdong Laboratory (Guangzhou), Guangzhou 511458, China; wzf@scbg.ac.cn (Z.W.)
* Correspondence: Mei Zhang (zhangmei@scbg.ac.cn); Zhengfeng Wang (wzf@scbg.ac.cn)

Table S1 The sequences of CrHsp20 protein, genomic DNA, CDS, and promoter region DNA, and the sequence information for the AtsHsps used in this study.

Table S1.xlsx

Table S2 Primer sequences used in this study.

Table S2.xlsx

Table S3 Summary of the *cis*-regulatory elements identified in the promoter regions of *CrHsp20* genes.

Table S3.xlsx

Table S4 The FPKM values of *CrHsp20*s for RNA-Seq assay of *C. rosea* tissues in this study.

Table S4.xlsx
